# Supplementary material for: Molecular phylogenetics, seed morphometrics, chromosome number evolution and systematics of European Elatine L. (Elatinaceae) species
Source: PeerJ. 2016 Dec 21;4:e2800. doi: 10.7717/peerj.2800 (PMC5180590; doi:10.7717/peerj.2800)
Supplement: Table S1 — The species selected for the initial screening were E. alsinastrum (outgroup: O), E. campylosperma (ingroup: I1), and E. hydropier (ingroup: I2). [file peerj-04-2800-s001.doc]

Table S1. The DNA regions screened for molecular variability in this study. The species selected for the initial screening were *E. alsinastrum* (outgroup: O), *E. campylosperma* (ingroup: I1), and *E. hydropier* (ingroup: I2).

| DNA region | encoded in | length | Aligned length | no. of variable characters | |  |
| --- | --- | --- | --- | --- | --- | --- |
|  |  |  |  | O vs. I1+I2 | I1 vs. I2 | ^1^source of primer and amplification information |
| *accD-psaI* | plastid | 1131–1144 | 1166 | 28 | 8 | Small & al. (1998) |
| At103 | nucleus | 429–432 | 436 | 43 | NA | Li & al. (2008) |
| *atpB* | plastid | 1100 | 1100 | 13 | 6 | Hoot & al. (1995) |
| Eif3E | nucleus | 671–679 | 680 | 36 | 3 | Li & al. (2008) |
| ITS | nucleus | 630–632 | 633 | 46 | 21 | Gulyás & al. (2005) |
| *matK* | plastid | 1065–1066 | 1066 | 18 | 5 | – |
| *nad6* | mitochondrion | 472 | 472 | NA | 1 | – |
| *rbcL* | plastid | 654 | 654 | 8 | 0 | – |
| *trnH-psbA* | plastid | 295–301 | 304 | 16 | 10 | Hamilton (1999) |
| *trnL-trnF* | plastid | 942–958 | 969 | 30 | 15 | Taberlet & al. (1991) |
| *ycf6-psbM* | plastid | 947–991 | 1023 | 37 | 13 | Shaw & al. (2005) |
| *psbM-trnD* | plastid | 521–537 | 549 | 11 | 5 | Shaw & al. (2005) |
| *psbJ-petA* | plastid | 570–591 | 594 | 21 | 10 | Shaw & al. (2007) |
