# Supplementary material for: Molecular phylogenetics, seed morphometrics, chromosome number evolution and systematics of European Elatine L. (Elatinaceae) species
Source: PeerJ. 2016 Dec 21;4:e2800. doi: 10.7717/peerj.2800 (PMC5180590; doi:10.7717/peerj.2800)
Supplement: Table S2 — Rows: given group; columns: predicted groups. All the measured populations were significantly different except the two E. hexandra samples. [file peerj-04-2800-s002.doc]

Table S2. Results of MANOVA *post-hoc* tests. Rows: given group; columns: predicted groups. All the measured populations were significantly different except the two *E. hexandra* samples.

|  | **alsKon** | **braLa** | **broMar** | **broSpn** | **calLa** | **camSar** | **gusGoz** | **gusLam** | **gusSic** | **gusSpn** | **hexJan** | **hexPoz** | **hunKon** | **hunWol** | **hydPol** | **hydTis** | **macSar** | **macSp** | **ortOul** | **triHun** | **triJa** |
| --- | --- | --- | --- | --- | --- | --- | --- | --- | --- | --- | --- | --- | --- | --- | --- | --- | --- | --- | --- | --- | --- |
| alsKon | - | p<0.001 | p<0.001 | p<0.001 | p<0.001 | p<0.001 | p<0.001 | p<0.001 | p<0.001 | p<0.001 | p<0.001 | p<0.001 | p<0.001 | p<0.001 | p<0.001 | p<0.001 | p<0.001 | p<0.001 | p<0.001 | p<0.001 | p<0.001 |
| braLa | p<0.001 | - | p<0.001 | p<0.001 | p<0.001 | p<0.001 | p<0.001 | p<0.001 | p<0.001 | p<0.001 | p<0.001 | p<0.001 | p<0.001 | p<0.001 | p<0.001 | p<0.001 | p<0.001 | p<0.001 | p<0.001 | p<0.001 | p<0.001 |
| broMar | p<0.001 | p<0.001 | - | p<0.001 | p<0.001 | p<0.001 | p<0.001 | p<0.001 | p<0.001 | p<0.001 | p<0.01 | p<0.001 | p<0.001 | p<0.001 | p<0.001 | p<0.001 | p<0.001 | p<0.001 | p<0.001 | p<0.001 | p<0.001 |
| broSpn | p<0.001 | p<0.001 | p<0.001 | - | p<0.001 | p<0.001 | p<0.001 | p<0.001 | p<0.001 | p<0.001 | p<0.001 | p<0.001 | p<0.001 | p<0.001 | p<0.001 | p<0.001 | p<0.001 | p<0.001 | p<0.001 | p<0.001 | p<0.001 |
| calLa | p<0.001 | p<0.001 | p<0.001 | p<0.001 | - | p<0.001 | p<0.001 | p<0.001 | p<0.001 | p<0.001 | p<0.001 | p<0.001 | p<0.001 | p<0.001 | p<0.001 | p<0.001 | p<0.001 | p<0.001 | p<0.001 | p<0.001 | p<0.001 |
| camSar | p<0.001 | p<0.001 | p<0.001 | p<0.001 | p<0.001 | - | p<0.001 | p<0.001 | p<0.001 | p<0.001 | p<0.001 | p<0.001 | p<0.001 | p<0.001 | p<0.001 | p<0.001 | p<0.001 | p<0.001 | p<0.001 | p<0.001 | p<0.001 |
| gusGoz | p<0.001 | p<0.001 | p<0.001 | p<0.001 | p<0.001 | p<0.001 | - | p<0.001 | p<0.001 | p<0.001 | p<0.001 | p<0.001 | p<0.001 | p<0.001 | p<0.001 | p<0.001 | p<0.001 | p<0.001 | p<0.001 | p<0.001 | p<0.001 |
| gusLam | p<0.001 | p<0.001 | p<0.001 | p<0.001 | p<0.001 | p<0.001 | p<0.001 | - | p<0.001 | p<0.001 | p<0.001 | p<0.001 | p<0.001 | p<0.001 | p<0.001 | p<0.001 | p<0.001 | p<0.001 | p<0.001 | p<0.001 | p<0.001 |
| gusSic | p<0.001 | p<0.001 | p<0.001 | p<0.001 | p<0.001 | p<0.001 | p<0.001 | p<0.001 | - | p<0.001 | p<0.001 | p<0.001 | p<0.01 | p<0.001 | p<0.001 | p<0.001 | p<0.001 | p<0.001 | p<0.001 | p<0.001 | p<0.001 |
| gusSpn | p<0.001 | p<0.001 | p<0.001 | p<0.001 | p<0.001 | p<0.001 | p<0.001 | p<0.001 | p<0.001 | - | p<0.001 | p<0.001 | p<0.001 | p<0.001 | p<0.001 | p<0.001 | p<0.001 | p<0.001 | p<0.001 | p<0.001 | p<0.001 |
| hexJan | p<0.001 | p<0.001 | p<0.01 | p<0.001 | p<0.001 | p<0.001 | p<0.001 | p<0.001 | p<0.001 | p<0.001 | - | 0.65 | p<0.001 | p<0.001 | p<0.001 | p<0.001 | p<0.001 | p<0.001 | p<0.001 | p<0.001 | p<0.01 |
| hexPoz | p<0.001 | p<0.001 | p<0.001 | p<0.001 | p<0.001 | p<0.001 | p<0.001 | p<0.001 | p<0.001 | p<0.001 | 0.65 | - | p<0.001 | p<0.001 | p<0.001 | p<0.001 | p<0.001 | p<0.001 | p<0.001 | p<0.001 | p<0.001 |
| hunKon | p<0.001 | p<0.001 | p<0.001 | p<0.001 | p<0.001 | p<0.001 | p<0.001 | p<0.001 | p<0.01 | p<0.001 | p<0.001 | p<0.001 | - | p<0.001 | p<0.001 | p<0.001 | p<0.001 | p<0.001 | p<0.001 | p<0.001 | p<0.001 |
| hunWol | p<0.001 | p<0.001 | p<0.001 | p<0.001 | p<0.001 | p<0.001 | p<0.001 | p<0.001 | p<0.001 | p<0.001 | p<0.001 | p<0.001 | p<0.001 | - | p<0.001 | p<0.001 | p<0.001 | p<0.001 | p<0.001 | p<0.001 | p<0.001 |
| hydPol | p<0.001 | p<0.001 | p<0.001 | p<0.001 | p<0.001 | p<0.001 | p<0.001 | p<0.001 | p<0.001 | p<0.001 | p<0.001 | p<0.001 | p<0.001 | p<0.001 | - | p<0.001 | p<0.001 | p<0.001 | p<0.001 | p<0.001 | p<0.001 |
| hydTis | p<0.001 | p<0.001 | p<0.001 | p<0.001 | p<0.001 | p<0.001 | p<0.001 | p<0.001 | p<0.001 | p<0.001 | p<0.001 | p<0.001 | p<0.001 | p<0.001 | p<0.001 | - | p<0.001 | p<0.001 | p<0.001 | p<0.001 | p<0.001 |
| macSar | p<0.001 | p<0.001 | p<0.001 | p<0.001 | p<0.001 | p<0.001 | p<0.001 | p<0.001 | p<0.001 | p<0.001 | p<0.001 | p<0.001 | p<0.001 | p<0.001 | p<0.001 | p<0.001 | - | p<0.001 | p<0.001 | p<0.001 | p<0.001 |
| macSp | p<0.001 | p<0.001 | p<0.001 | p<0.001 | p<0.001 | p<0.001 | p<0.001 | p<0.001 | p<0.001 | p<0.001 | p<0.001 | p<0.001 | p<0.001 | p<0.001 | p<0.001 | p<0.001 | p<0.001 | - | p<0.001 | p<0.001 | p<0.001 |
| ortOul | p<0.001 | p<0.001 | p<0.001 | p<0.001 | p<0.001 | p<0.001 | p<0.001 | p<0.001 | p<0.001 | p<0.001 | p<0.001 | p<0.001 | p<0.001 | p<0.001 | p<0.001 | p<0.001 | p<0.001 | p<0.001 | - | p<0.001 | p<0.001 |
| triHun | p<0.001 | p<0.001 | p<0.001 | p<0.001 | p<0.001 | p<0.001 | p<0.001 | p<0.001 | p<0.001 | p<0.001 | p<0.001 | p<0.001 | p<0.001 | p<0.001 | p<0.001 | p<0.001 | p<0.001 | p<0.001 | p<0.001 | - | p<0.01 |
| triJa | p<0.001 | p<0.001 | p<0.001 | p<0.001 | p<0.001 | p<0.001 | p<0.001 | p<0.001 | p<0.001 | p<0.001 | p<0.01 | p<0.001 | p<0.001 | p<0.001 | p<0.001 | p<0.001 | p<0.001 | p<0.001 | p<0.001 | p<0.01 | - |
