# Supplementary material for: Molecular phylogenetics, seed morphometrics, chromosome number evolution and systematics of European Elatine L. (Elatinaceae) species
Source: PeerJ. 2016 Dec 21;4:e2800. doi: 10.7717/peerj.2800 (PMC5180590; doi:10.7717/peerj.2800)
Supplement: Table S3 — Correct assignments are indicated with grey. The algorithm could assign correctly 64.1% of the specimens. Classification success varies between 20% and 96%. [file peerj-04-2800-s003.doc]

Table S3. Jackknife classification of the measured individuals. Correct assignments are indicated with grey. The algorithm could assign correctly 64.1% of the specimens. Classification success varies between 20% and 96%.

|  | **alsKon** | **braLa** | **broMar** | **broSpn** | **calLa** | **camSar** | **gusGoz** | **gusLam** | **gusSic** | **gusSpn** | **hexJan** | **hexPoz** | **hunKon** | **hunWol** | **hydPol** | **hydTis** | **macSar** | **macSp** | **ortOul** | **triHun** | **triJa** |
| --- | --- | --- | --- | --- | --- | --- | --- | --- | --- | --- | --- | --- | --- | --- | --- | --- | --- | --- | --- | --- | --- |
| alsKon | 37 | 0 | 0 | 0 | 0 | 0 | 0 | 0 | 0 | 0 | 0 | 4 | 0 | 0 | 0 | 0 | 0 | 0 | 0 | 2 | 3 |
| braLa | 0 | 21 | 13 | 7 | 0 | 0 | 0 | 0 | 0 | 0 | 6 | 0 | 0 | 0 | 0 | 0 | 1 | 2 | 0 | 0 | 0 |
| broMar | 1 | 6 | 31 | 2 | 0 | 0 | 0 | 0 | 0 | 0 | 3 | 2 | 0 | 0 | 0 | 0 | 0 | 1 | 0 | 3 | 1 |
| broSpn | 0 | 6 | 4 | 39 | 0 | 0 | 0 | 0 | 0 | 0 | 0 | 1 | 0 | 0 | 0 | 0 | 0 | 0 | 0 | 0 | 0 |
| calLa | 0 | 0 | 0 | 0 | 32 | 0 | 0 | 2 | 1 | 7 | 0 | 0 | 2 | 6 | 1 | 0 | 0 | 0 | 0 | 0 | 0 |
| camSar | 0 | 0 | 0 | 0 | 0 | 35 | 0 | 0 | 0 | 1 | 0 | 0 | 0 | 0 | 4 | 9 | 0 | 0 | 0 | 0 | 0 |
| gusGoz | 0 | 0 | 0 | 0 | 1 | 0 | 40 | 2 | 3 | 0 | 0 | 0 | 2 | 0 | 0 | 0 | 2 | 0 | 0 | 0 | 0 |
| gusLam | 0 | 0 | 0 | 0 | 3 | 0 | 2 | 32 | 1 | 4 | 0 | 0 | 6 | 1 | 1 | 0 | 0 | 0 | 0 | 0 | 0 |
| gusSic | 0 | 0 | 0 | 0 | 0 | 0 | 3 | 3 | 22 | 5 | 0 | 0 | 8 | 8 | 1 | 0 | 0 | 0 | 0 | 0 | 0 |
| gusSpn | 0 | 0 | 0 | 0 | 4 | 0 | 0 | 1 | 4 | 26 | 0 | 0 | 1 | 12 | 2 | 0 | 0 | 0 | 0 | 0 | 0 |
| hexJan | 0 | 2 | 2 | 0 | 0 | 0 | 0 | 0 | 0 | 0 | 10 | 10 | 0 | 0 | 0 | 0 | 2 | 3 | 0 | 0 | 3 |
| hexPoz | 2 | 1 | 3 | 0 | 0 | 0 | 0 | 0 | 0 | 0 | 8 | 17 | 0 | 0 | 0 | 0 | 3 | 4 | 0 | 8 | 4 |
| hunKon | 0 | 0 | 0 | 0 | 1 | 0 | 3 | 7 | 9 | 0 | 0 | 0 | 28 | 2 | 0 | 0 | 0 | 0 | 0 | 0 | 0 |
| hunWol | 0 | 0 | 0 | 0 | 0 | 0 | 0 | 3 | 3 | 8 | 0 | 0 | 1 | 35 | 0 | 0 | 0 | 0 | 0 | 0 | 0 |
| hydPol | 0 | 0 | 0 | 0 | 0 | 3 | 0 | 1 | 0 | 1 | 0 | 0 | 0 | 0 | 40 | 5 | 0 | 0 | 0 | 0 | 0 |
| hydTis | 0 | 0 | 0 | 0 | 0 | 4 | 0 | 0 | 0 | 0 | 0 | 0 | 0 | 0 | 0 | 46 | 0 | 0 | 0 | 0 | 0 |
| macSar | 0 | 0 | 0 | 0 | 0 | 0 | 5 | 0 | 0 | 0 | 3 | 0 | 0 | 0 | 0 | 0 | 30 | 7 | 4 | 0 | 1 |
| macSp | 0 | 1 | 0 | 0 | 0 | 0 | 0 | 0 | 0 | 0 | 1 | 2 | 0 | 0 | 0 | 0 | 7 | 39 | 0 | 0 | 0 |
| ortOul | 1 | 0 | 1 | 0 | 0 | 0 | 0 | 0 | 0 | 0 | 0 | 2 | 0 | 0 | 0 | 0 | 1 | 1 | 39 | 1 | 4 |
| triHun | 4 | 0 | 1 | 1 | 0 | 0 | 0 | 0 | 0 | 0 | 0 | 5 | 0 | 0 | 0 | 0 | 0 | 0 | 0 | 35 | 4 |
| triJa | 1 | 0 | 1 | 0 | 0 | 0 | 0 | 0 | 0 | 0 | 2 | 6 | 0 | 0 | 0 | 0 | 3 | 0 | 4 | 14 | 19 |
